# Clinical Misdiagnosis of COVID-19 Infection with Confusing Clinical Course

**DOI:** 10.1155/2021/6629966

**Published:** 2021-03-31

**Authors:** Hamid Eshaghi, Vahid Ziaee, Mahmood Khodabande, Moeinadin Safavi, Elmira Haji Esmaeil Memar

**Affiliations:** ^1^Department of Pediatric Infectious Disease, Children's Medical Center, Tehran University of Medical Sciences, Tehran, Iran; ^2^Pediatric Rheumatology Research Group, Rheumatology Research Center, Tehran University of Medical Sciences, Tehran, Iran; ^3^Department of Infectious Diseases, Pediatric‘s Center of Excellence, Children‘s Medical Center, Tehran University of Medical Sciences, Tehran, Iran; ^4^Pathology Department, Children's Medical Center, Tehran University of Medical Sciences, Tehran, Iran; ^5^Pediatric Department, Pediatric Center of Excellence, Children's Medical Center, Tehran University of Medical Sciences, Tehran, Iran

## Abstract

**Background:**

Similarities in the febrile course and other manifestations of some diseases may lead to clinical misdiagnosis of COVID-19 infection. Here, we report a case in a young child with a potentially confusing clinical course. *Case Presentation*. A 29-month-old boy presented with a 2-month history of fever. His PCR test for COVID-19 was positive, and there was pleural effusion plus positive findings in the lower left lobe of the lung on computed tomography scan. Mid-sized splenomegaly was found on abdominal ultrasound, and laboratory tests disclosed pancytopenia. In light of the atypical lymphocyte counts in laboratory tests, he underwent bone marrow aspiration. The suggested diagnosis was hemophagocytic lymphohistiocytosis, and prednisolone was initiated. Subsequently, Leishman-Donovan bodies were seen in the bone marrow aspirate, and treatment was started with amphotericin, which led to clinical improvement.

**Conclusion:**

In cases with vague clinical symptoms in tropical countries where other infectious diseases occur, possible simultaneous infection should be considered even during a pandemic. Familiarity with the possible differential diagnoses and appropriate, step-by-step consideration to rule out other possible causes are needed in all situations, and the coexistence of infectious disease should be considered in evaluating the clinical conditions of patients in tropical countries.

## 1. Introduction

The current COVID-19 pandemic, which originated in China in December 2019, has rapidly spread throughout the world, resulting in a life-threatening pandemic [1, 2]. Members of this virus family have been known since the 1960s, but the current COVID-19 pandemic has heightened global concerns regarding other febrile diseases that predominantly affect the respiratory system [3, 4]. In addition, gastrointestinal and hepatic involvement in COVID-19 can mimic the clinical manifestations of the other infectious diseases [5].

During the COVID-19 pandemic, coexistence with other diseases is not unusual, and misdiagnoses may be inevitable. These issues are more common in tropical countries affected by infectious disease with signs and symptoms on presentation similar to COVID-19, and the resulting delays in diagnosis and treatment can lead to problems for some patients, especially in the pediatric population. In our region, leishmaniasis is one such tropical disease that can present diagnostic challenges. The visceral type is characterized by long-term fever, anemia, organomegaly, and lymphadenopathy [6, 7]. Similarities in the febrile course and other manifestations may lead to clinical misdiagnosis of COVID-19 disease. One of these differential diagnoses according to the clinical manifestation of our patient is hemophagocytic lymphohistiocytosis (HLH) disease. HLH is a rare condition of pathologic immune activation, of which its secondary form may appear as a secondary process of malignancies, infections, metabolic disorders, and rheumatological or autoimmune disorders. Infectious agents are mostly cytomegalovirus (CMV) and Epstein–Barr virus (EBV) [6].

Here, we report a case in a young child with a potentially confusing clinical course that had hemophagocytic lymphohistiocytosis secondary to visceral leishmaniasis.

## 2. Case Presentation

**Figure 1:**
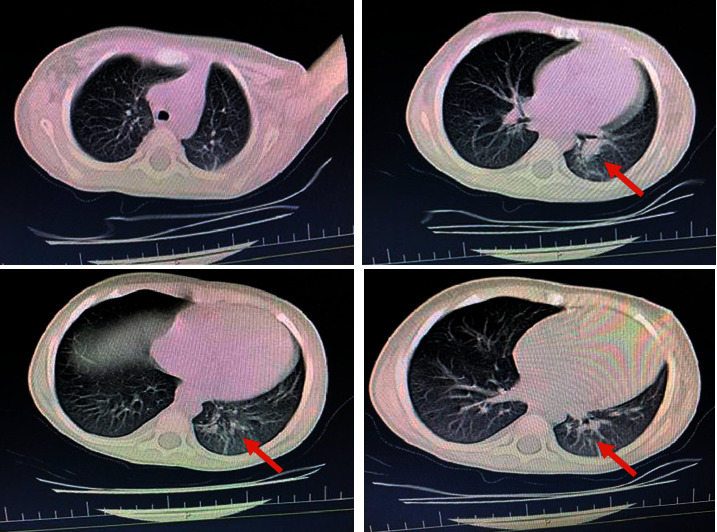
Spiral CT scan of chest without contrast.

A 29-month-old boy presented with a 2-month history of fever. After evaluation at several different out-patient services, he was admitted to our hospital. His PCR test for COVID-19 was positive, and there was pleural effusion plus positive findings in the lower left lobe of the lung on a computed tomography scan of his chest (Figure 1).

In addition, mid-sized splenomegaly (130 mm) was found on the first abdominal ultrasound.

As shown in Table 1, baseline results of laboratory tests disclosed pancytopenia.

The patient was discharged from the first hospital and they gave them instructions to take the boy to our tertiary healthcare center.

On admission, hydroxyl-chloroquine was administered to complete the treatment course for COVID-19. Fever and pancytopenia were treated with vancomycin plus cefepime.

The liver had normal echoes on the second ultrasonography and measured 84 mm, and the gallbladder wall was thickened. Other ultrasonographic findings were normal, except for a parenchymal cyst in the upper kidney pole.

**Figure 2:**
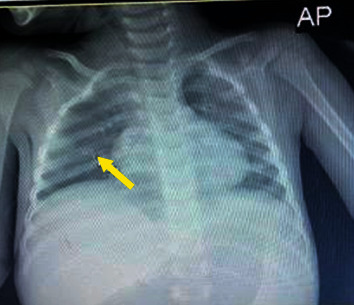
Chest radiography AP view.

According to an AP view of chest radiography, there was ground-glass consolidation behind the cardiac shadow, although heart size was normal. In addition, there was a splenomegaly (Figure 2).

Echocardiography showed very mild pulmonary embolism and patent foramen ovale.

In addition, laboratory tests showed abnormal liver function, pancytopenia, hypertriglyceridemia, increased ferritin level, and decreased fibrinogen (Table 1).

**Figure 3:**
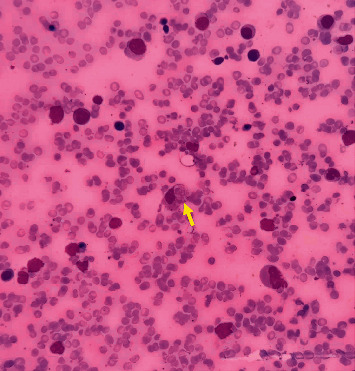
Bone marrow aspiration smear: a histiocyte with engulfed red blood cell and a Leishman Donovan like body (Giemsa x400).

In order to rule out other differential diagnoses, other laboratory tests were performed (Table 2). In light of the atypical lymphocyte counts in the first laboratory tests, he underwent bone marrow aspiration which was normal. Also, the direct antiglobulin test was positive with a titer of 1 : 3200. Subsequently, Leishman Donovan bodies and hemophagocytic cells were seen in bone marrow aspiration (Figure 3), and treatment was started with amphotericin, which led to clinical improvement. Then, the suggested diagnosis was hemophagocytic lymphohistiocytosis secondary to visceral leishmaniasis, and prednisolone was initiated.

## 3. Discussion

In non-COVID-19 pandemic conditions, the primary diagnosis in patients such as the young boy described here would undoubtedly be leishmaniasis, a tropical disease prevalent in Iran. However, because most patients with visceral leishmaniasis are asymptomatic, the presence of certain clinical manifestations may be confusing to some degree [8, 9]. Simultaneous infection by other pathogens has been reported (for example) for HIV [10]. An important point to consider at the present time is the role of transmission of the infectious agent by patients who remain undiagnosed [11, 12]. Cortes et al. [13] reported coinfection by *Leishmania braziliensis* and *Streptococcus pneumoniae* in patients with multiple skin lesions. A prompt definite diagnosis can lead sooner to appropriate treatment and better outcomes.

Our patient may be the first to be reported with coinfection by SARS-CoV-2 and *Leishmania* sp. Recently, Zhou et al. reported coinfection by the COVID-19 virus and bacterial and fungal disease agents [14]. These authors noted the importance of detecting coinfection for both therapeutic and epidemiological reasons. Unfortunately, patients with coinfection usually have a worse disease course and a poor prognosis [15]. Coinfection with other pathogens can alter the susceptibility to other important pathogens via effects on the host's immune responsiveness [16]. For example, helminths coinfection may modulate COVID-19 severity in tropical regions [17]. Consideration of the multiple interactions in coinfections will allow clinicians to better predict the response to medical interventions and environmental changes [18].

As shown by El Hassan et al. [19], post-kala-azar dermal leishmaniasis is a complication in patients with visceral leishmaniasis which can further complicate the clinical picture and result in misdiagnosis. However, in patients with solely visceral involvement, an accurate diagnosis is more feasible. Singh and Sundar [20] noted the problems connected with overlooking a single disease subtype that may manifest with ambiguous signs or symptoms in patients presenting with complaints that point to several diagnostic possibilities. Health risks arise when some of these patients with confusing or mild-appearing clinical presentations are misdiagnosed.

The clinical presentations of all forms of visceral leishmaniasis change from time to time, and this can be a source of confusion, especially in patients with common or simultaneous microbial diseases [21]. Gawade et al. [22] reported a 20-year-old agricultural laborer with a history of recurrent febrile episodes, progressive weakness, and abdominal discomfort associated with anorexia for 6 months followed by petechial hemorrhages over various parts of the body. Such manifestations may help to better distinguish between different entities in the differential diagnosis.

In nontropical regions, other obstacles to a prompt, accurate diagnosis can arise because visceral leishmaniasis is hard to recognize and relatively new in these countries, making misdiagnosis more common and thus delaying treatment or leading to inappropriate treatment [23]. It may be misdiagnosed as solely involvement of organs such as the liver [24]. Caution is particularly needed in these settings because of fatal cases of visceral leishmaniasis that cannot be prevented [25].

In conclusion, patients with vague clinical symptoms in tropical countries where other infectious diseases are prevalent should be carefully evaluated to identify possible simultaneous infections, even in the context of an ongoing epidemic or a pandemic. Familiarity with the possible differential diagnoses and appropriate, step-by-step consideration to rule out other possible causes are needed in all situations, and the coexistence of more than one infectious disease should be considered in evaluating the clinical conditions of patients in tropical countries.

## Figures and Tables

**Table 1:** Laboratory findings of the patient during admission.

Parameter	Baseline	Day 1	Day 2	Day 3	Day 4	Day 5	Day 6
Uric acid (3–5.5 mg/dl)	2.8						
Cholesterol (130–200 mg/dl)	467			390			456
TG (40–160 U/L)	735		331		340		296
AST (10–40 U/L)	103	31	143	62	83	45	31
ALT (10–40 U/L)	121	49	91	45	38		21
Bili total (1–1.2 mg/dl)	7						2.4
Bili direct (0.1–0.4 mg/dl)	0.6						0.5
LDH (5–850 IU/L)	770						729
Albumin (3.5–5.2 gr/dl)	3.8	2.9	2.5	3.3	3.1	4	3.5
Total protein (5.7–8 gr/dl)	4.6		4.5		5.3		5.8
CRP (<6 mg/L)	12			12			11
ESR (<15 mm/hours)	28						
Procalcitonin (<0.05 ng/ml)	1.6						0.1
CPK (24–195 U/L)	17						
Fibrinogen (150–350 mg/dl)	245			180			147
WBC ((4–10) × 103/UL)	3.41		2.17	1.74		2.44	2.13
PMN ((2–7) × 103/UL)	16.6		14.7	26		6.6	3.8
Lymph ((0.8–4) × 103/UL)	72		70	68		61	69.6
RBC ((3.5–5.5) × 103/UL)	2.8	1.94	2.84	2.77	2.33	2.96	2.43
Hb (11–16 gr/dl)	7.6		5.2	7.8		8	6.6
Ferritin (30–220 ng/ml)	7720	6770	3480	24320	18800	27760	19360
Plt ((150–450) × 103/UL)	37		32	23		13	20
PT (11–13.8 second)	14						12.5
PTT (11–13.8 second)	33						36
INR (2, 3)	1.2						1
CD4 (31–64%)	52			50			12
CD8 (8–41%)	21			28			79
C3 (81–170 mg/dl)	149	
C4 (9.2–34 mg/dl)	63						
CH30 (70–150)	140						
Antitetanus (<0.1 IU/ml)	0.24						
Antidiphtheria (<0.1 IU/ml)	0.07						
NBT (90–100)	100%						
DAT	1/3200						
SARS-CoV-2 (COVID-19) IgG (<0.9)	0.5						
SARS-CoV-2 (COVID-19) IgM (<0.9)	0.1						

**Table 2:** Other laboratory findings to rule out different differential diagnosis.

Blood culture	Negative	
Peripheral blood smear	Leishman body	
Flow cytometry	Blasts 2%	
Urine culture	Negative	
Bone marrow culture	Negative	
Wright	Negative	
Coombs-Wright	Negative	
2ME	Negative	
Widal	Negative	
Flow cytometry	Normal	
Kala-azar IgG	Negative	
Kala-azar IgM	Negative	
HIV Ab (<1 nonreactive)	0.1	
IgG (Elisa) (295–1156) mg/dl	205	
IgM (Elisa) (37–184) mg/dl	18	
EBV-CMV	Negative	
EBV-IgG (<0.8 nonreactive) U/ml	4.7	
EBV-IgM (<9 nonreactive) U/ml	0.1	
CMV-IgG (<6 nonreactive) U/ml	0.1	
CMV-IgM (<0.8 nonreactive) U/ml	0.1	
Malaria-*Borrelia*	Negative	
EMG	Negative	

Pleural fluid analysis		
Volume	5	
Color	Red	
Appearance	Turbid	
WBC	20	90% (lymphocytes)
		10% (PMN)
RBC	60000	
Direct smear	Bacteria were not seen	
Pleural fluid culture	No growth after 72 hr	

## Data Availability

The datasets used in the current study are available from the corresponding author on reasonable request.
